# Suppression of TGFβ-Induced Epithelial-Mesenchymal Transition Like Phenotype by a PIAS1 Regulated Sumoylation Pathway in NMuMG Epithelial Cells

**DOI:** 10.1371/journal.pone.0013971

**Published:** 2010-11-12

**Authors:** Stuart J. Netherton, Shirin Bonni

**Affiliations:** Department of Biochemistry and Molecular Biology, Southern Alberta Cancer Research Institute, University of Calgary, Calgary, Alberta, Canada; University Medical Center Maastricht, Netherlands

## Abstract

Epithelial-mesenchymal-transition (EMT) is a fundamental cellular process that is critical for normal development and tumor metastasis. The transforming growth factor beta (TGFβ) is a potent inducer of EMT like effects, but the mechanisms that regulate TGFβ-induced EMT remain incompletely understood. Using the widely employed NMuMG mammary epithelial cells as a model to study TGFβ-induced EMT, we report that TGFβ downregulates the levels of the SUMO E3 ligase PIAS1 in cells undergoing EMT. Gain and loss of function analyses indicate that PIAS1 acts in a SUMO ligase dependent manner to suppress the ability of TGFβ to induce EMT in these cells. We also find that TGFβ inhibits sumoylation of the PIAS1 substrate SnoN, a transcriptional regulator that antagonizes TGFβ-induced EMT. Accordingly, loss of function mutations of SnoN sumoylation impair the ability of SnoN to inhibit TGFβ-induced EMT in NMuMG cells. Collectively, our findings suggest that PIAS1 is a novel negative regulator of EMT and reveal that inhibition of the PIAS1-SnoN sumoylation pathway represents a key mechanism by which TGFβ induces EMT, with important implications in normal development and tumor metastasis.

## Introduction

Epithelial-mesenchymal transition (EMT) is a fundamental biological process that is critical for proper tissue and organ morphogenesis in the developing organism [1]. EMT also is important for wound healing [1]. EMT is reactivated in pathological conditions including fibrotic and neoplastic diseases, and importantly EMT contributes to tumor metastasis [1], [2], [3], [4]. Epithelial cells are characterized by apical-basal polarity that is maintained by specialized junctions including the apical tight junctions and basolateral adherens junctions as well as by an organized cytoskeletal architecture. Thus, EMT induces a morphological alteration in epithelial cells from an apical-basal polarized to a spindle-like non-polarized phenotype. EMT comprises the loss of epithelial cell polarity and cell-cell adhesion due to changes in cytoskeletal architecture and cell junctional proteins [2], [3], [5], [6]. Consistent with these morphological alterations, cells undergoing EMT show a change in the actin cytoskeleton from cortical F-actin type to stress fiber-like. EMT also induces downregulation or mislocalization of the epithelial marker and adherens junctional protein E-cadherin [7]. E-cadherin exerts a central role in epithelial homeostasis and its loss at the cell-cell junctions leads to reduced expression and/or organization of other epithelial markers including zona occludins-1 [8]. Reduction in E-cadherin levels at the sites of cell-cell attachments is considered to be a hallmark of EMT [9], [10], [11]. In addition, downregulation of E-cadherin is a predictive marker of invasiveness and metastatic potential of many forms of cancer, including breast and gastric tumors [12], [13], [14], [15].

The transforming growth factor beta (TGFβ) family of proteins plays pleiotropic and essential roles in normal development and homeostasis [16], [17]. A key function of TGFβ is its ability to induce EMT [1], [5], [6], [18]. TGFβ-induced EMT plays critical roles in development and wound healing, and contributes to the ability of TGFβ to promote tumor progression observed at later stages of many malignancies including mammary, prostate, and colorectal carcinomas [5], [19], [20], [21], [22].

TGFβ initiates signaling in responsive cells by forming an activated heteromeric complex with specific transmembrane TGFβ type I and II serine/threonine kinase receptors [23], [24], [25], [26]. The type II receptor kinase phosphorylates and activates the type I receptor, which in turn induces the activation of the canonical intracellular Smad signaling pathway [27], [28], [29], [30], [31]. The Smad proteins, which are transcription factors, are required for the ability of TGFβ to induce EMT. In particular, the Smad proteins induce the expression of other transcription factors, including Snail and Zeb1, that are thought to repress the expression of the E-cadherin gene [22]. Although the mechanisms that mediate TGFβ induction of EMT are beginning to be elucidated, how the function of TGFβ in EMT is regulated has remained unexplored.

PIAS1 [protein inhibitor of activated STAT (signal transducer and activator of transcription) 1] was originally identified based on its ability to interact with and inhibit STAT1 binding to DNA [32], [33]. Later studies showed that PIAS1 is a SUMO E3 ligase [34], [35], [36]. Sumoylation involves the covalent attachment of the protein SUMO (small ubiquitin like modifier) to ε-amino group in lysine residues of target substrates. Sumoylation is performed by the sequential action of three sets of enzymes [37]. In the first step, an E1 enzyme covalently binds a SUMO molecule in an ATP-dependent fashion. The SUMO molecule is transferred next to the SUMO E2 conjugating enzyme Ubc9. A SUMO E3 ligase binds to Ubc9 and specific substrates, and thereby facilitates the transfer of a SUMO molecule from Ubc9 to specific lysine residues within substrates. As a SUMO E3 ligase, PIAS1 enhances the sumoylation of transcriptional regulators, including the transcriptional modulator SnoN, which plays a critical role in TGFβ responses [38], [39]. However, the functional significance of PIAS1 in TGFβ-induced EMT induction has remained unknown.

In this study, we uncover a novel mechanism that regulates EMT. We show that TGFβ reduces the level of the SUMO E3 ligase PIAS1 in cells undergoing EMT. Loss and gain of function experiments suggest that PIAS1 antagonizes EMT. We also find that TGFβ leads to inhibition of sumoylation of the PIAS1 substrate SnoN during EMT. Loss of function experiments demonstrate that sumoylation contributes to the ability of SnoN to inhibit EMT. Thus, our study identifies the SUMO E3 ligase PIAS1 as a novel regulator of TGFβ-induced EMT.

## Results

### TGFβ downregulates the SUMO ligase PIAS1 during EMT

**Figure 1:**
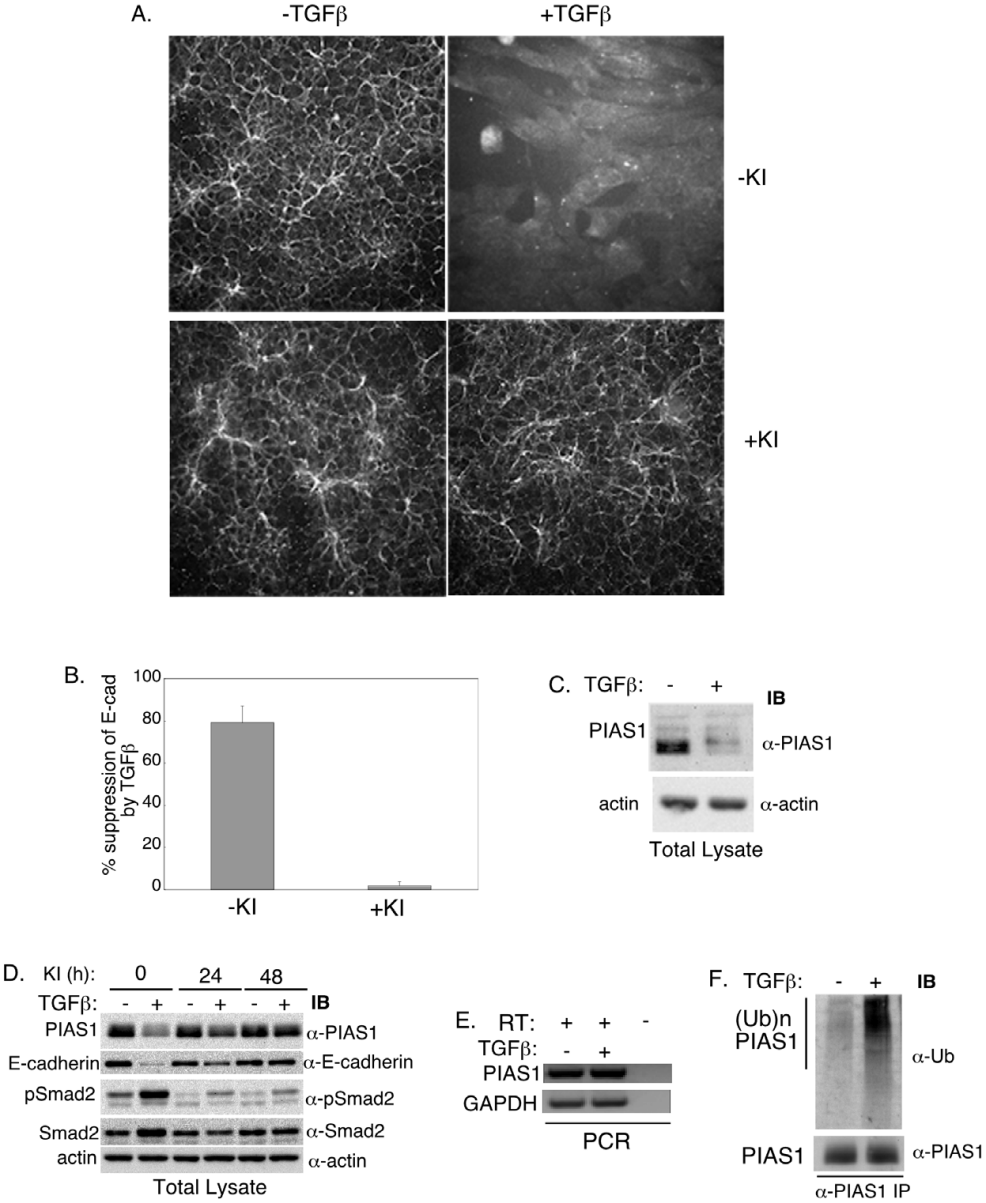
TGFβ downregulates the SUMO E3 ligase PIAS1 in cells undergoing EMT. A) E-cadherin immunofluorescence images of NMuMG cells, left untreated (− TGFβ), or incubated with TGFβ (+ TGFβ), the TGFβ-receptor type I kinase (TβRI) inhibitor SB431542 (+ KI), alone or together for 48 h. B) Each column is the mean (± SEM, n = 3 experiments) of percent suppression of cellular E-cadherin by TGFβ relative to that of the basal for each of −KI and +KI (see MATERIALS and METHODS). TGFβ induces EMT as indicated by nearly 90% reduction in the level of the epithelial marker E-cadherin in cells. The KI blocks TGFβ reduction of E-cadherin and hence induction of EMT. C) TGFβ decreases endogenous PIAS1 levels in NMuMG cells undergoing EMT. Lysates of NMuMG cells left untreated or incubated with TGFβ (48 h) were subjected to PIAS1 (α-PIAS1) and actin (α-actin) immunoblotting (IB). D) TβRI-signaling is required for downregulation of PIAS1. Lysates of NMuMG cells left untreated or incubated with TGFβ (48 h), KI (for 48 h or for the last 24 h), alone or together, were subjected to PIAS1, E-cadherin (α-E-cadherin), Smad2 (α-Smad2) and phospho-Smad2 (α-pSmad2) immunoblotting. TGFβ increases phospho-Smad2 while reducing the levels of PIAS1 and E-cadherin. The KI blocks the ability of TGFβ to downregulate PIAS1 and E-cadherin. E) TGFβ does not reduce the levels of PIAS1 mRNA. RT-PCR of RNA extracts from NMuMG cells incubated without or with TGFβ for 48 h to analyze mRNA levels of PIAS1 and the internal control GAPDH (see MATERIALS and METHODS). F) TGFβ enhances endogenous PIAS1 ubiquitination in NMuMG cells. Lysates of cells treated with the proteasome inhibitor MG132 (last 24 h of a 48 h culture), TGFβ (48 h), alone or together were subjected to PIAS1 immunoprecipitation followed by sequential ubiquitin and PIAS1 immunoblotting. Scans in C to F are representative of experiments that were carried out at least 3 independent times.

To identify novel regulators of EMT, we employed mouse mammary epithelial NMuMG cells, widely used for studies of EMT [40], [41], [42]. We first confirmed that TGFβ induced EMT in these cells. NMuMG cells that were untreated or incubated for 2 days with TGFβ in the absence or presence of TGFβ type I receptor kinase (TβRI) inhibitor SB431542 were assessed for EMT [43], [44]. A key molecular hallmark of EMT is loss or mislocalization of the epithelial cell-cell junction protein E-cadherin. We used an indirect immunofluorescence approach to visualize the localization and quantify the levels of E-cadherin using the Cellomics Kinetic Scan Reader (KSR) and associated SpotDetector bioapplication [45]. TGFβ led to marked reduction of E-cadherin in NMuMG cells, and the TβRI inhibitor completely blocked this effect of TGFβ (Figures 1A and 1B). We also monitored actin reorganization from cortical to stress fiber type and cell shape change from cuboidal to fibroblastic like, as additional alterations associated with EMT. We used fluorescently labeled phalloidin and the whole cell dye stain CMFDA to visualize changes in actin reorganization and cell shape, respectively. TGFβ stimulation triggered actin stress fiber formation and fibroblastic phenotype that were blocked by TβRI inhibitor (Figures S1A and S1B). Collectively, these data confirm that TGFβ induces EMT in these cells.

Next, we used a candidate approach to identify potential novel regulators of TGFβ-induced EMT. The SUMO E3 ligase PIAS1 can regulate TGFβ signaling but the potential role of PIAS1 in EMT remained unidentified. To determine the role of PIAS1 in TGFβ-induced EMT, we first measured the levels of PIAS1 in NMuMG cells that were left untreated or that were incubated with TGFβ for 2 days. Surprisingly, we found that TGFβ dramatically reduced the levels of PIAS1 in NMuMG cells (Figure 1C). The TβRI inhibitor blocked the ability of TGFβ to decrease the level of PIAS1 as well as that of the epithelial marker E-cadherin (Figure 1D). We confirmed that TβRI inhibitor blocked TGFβ-induced phosphorylation of Smad2 (Figure 1D). Next, we determined if the decrease in the levels of PIAS1 in cells undergoing EMT was at the level of transcription. However, TGFβ had little or no effect on the levels of PIAS1 mRNA (Figure 1E). We then considered the possibility that TGFβ-dependent reduction in the levels of PIAS1 might be due to an increase in PIAS1 ubiquitination and consequent degradation. To test this idea, we incubated control and TGFβ-treated NMuMG cells with the proteasome inhibitor MG132 to protect polyubiquitinated proteins from proteasomal degradation. We found that TGFβ induced the polyubiquitination of PIAS1 in NMuMG cells (Figure 1F). These data suggest that TGFβ reduces the level of PIAS1 via the ubiquitin-proteasome pathway in cells undergoing EMT.

PIAS1 is a member of the PIAS family of SUMO E3 ligases that also includes PIAS2, PIAS3, and PIAS4 [46]. We therefore also determined effect of TGFβ on the levels of PIAS 2, 3 and 4 mRNA and protein in NMuMG cells (see Materials and Methods S1). We found that PIAS2, PIAS3 and PIAS4 mRNAs were expressed in control NMuMG cells as determined by RT-PCR analyses, and their levels were not altered upon TGFβ treatment (Figure S1C). Immunoblotting analyses revealed that PIAS3 and PIAS4 were expressed in NMuMG cells but there was little or no expression of PIAS2 in these cells (Figure S1D). In contrast to TGFβ-induced downregulation of PIAS1 protein in NMuMG cells (Figure 1C), TGFβ did not appreciably alter the levels of the PIAS3 and PIAS4 proteins (Figure S1D). Together, these data suggest that TGFβ may selectively induce the downregulation of PIAS1 among the PIAS proteins.

### PIAS1 acts in a SUMO ligase dependent manner to inhibit EMT

**Figure 2:**
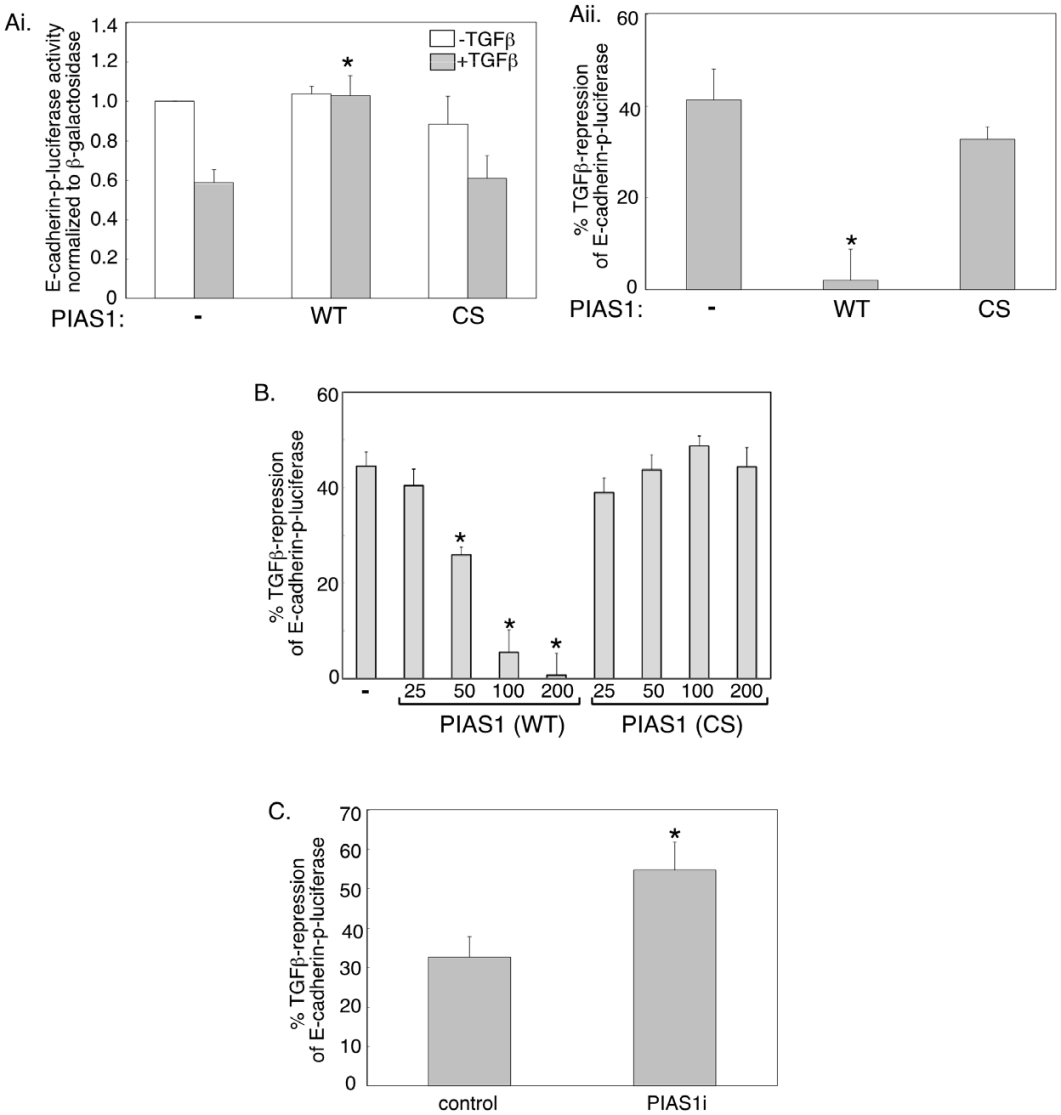
PIAS1 inhibits the ability of TGFβto suppress E-cadherin promoter activity. A) PIAS1 decreases TGFβ inhibition of E-cadherin promoter activity in a SUMO E3 ligase-dependent manner. Lysates of NMuMG cells transfected with the E-cadherin promoter driven luciferase (E-cadherin-p-luciferase) reporter and the β-galactosidase (β-gal) expression construct together with an empty expression vector (−), or one encoding wild type PIAS1 (WT) or SUMO E3 ligase mutant PIAS1 (CS), and left untreated or incubated with 100 pM TGFβ for 48 h, were subjected to luciferase and β-galactosidase assays (see MATERIALS and METHODS). Ai) Each column is the mean (± SEM, n = 5) of β-galactosidase normalized luciferase data relative to that of the basal (− TGFβ) vector control. Aii) Each column is the mean (± SEM, n = 5) of the percent repression of luciferase activity by TGFβ relative to that of the basal (− TGFβ) of the corresponding transfection as shown in Ai (see MATERIALS and METHODS). B) PIAS1 reversal of TGFβ-inhibition of the E-cadherin promoter activity is dose-dependent. Lysates of untreated or TGFβ-treated cells transfected with E-cadherin-p-luciferase reporter and β-gal plasmid together with an empty vector or increasing concentrations of plasmids containing PIAS1 (WT) or PIAS1 (CS) were subjected to luciferase and β-galactosidase assays. Each column is the mean (± SEM, n = 6) of percent suppression of E-cadherin luciferase activity by TGFβ. C) Endogenous PIAS1 knockdown enhances TGFβ suppression of E-cadherin reporter expression. Lysates of untreated or TGFβ treated NMuMG cells transiently transfected with the E-cadherin-p-luciferase reporter and β-gal construct together with a control vector (control) or one encoding short hairpin RNAs to knockdown endogenous PIAS1 (PIAS1i), were subjected to luciferase and β-galactosidase assays. Each column is the mean (± SEM, n = 6) of percent suppression of E-cadherin luciferase activity by TGFβ from six independent experiments. * indicates statistical significant difference (P<0.05) as compared to the corresponding TGFβ treatment vector control.

The finding that TGFβ reduces the level of PIAS1 in cells undergoing EMT led us to characterize the function of PIAS1 in EMT. We first tested the effect of expression of exogenous PIAS1 on an E-cadherin promoter driven luciferase reporter gene, whose repression represents a reliable readout of EMT [47], [48]. We confirmed that TGFβ suppressed expression of the E-cadherin luciferase reporter gene in NMuMG cells (Figures 2Ai and 2Aii). Expression of exogenous PIAS1 inhibited the ability of TGFβ to repress the E-cadherin reporter without affecting the basal level of the E-cadherin reporter in NMuMG cells (Figure 2Ai). The cysteine-rich RING type zinc finger domain in PIAS1 is required for its interaction with the E2 SUMO conjugating Ubc9 and hence for the E3 SUMO ligase activity of PIAS1 [35]. We tested the effect of expression of a mutant PIAS1 protein, in which Cysteine 350 within the zinc finger domain was converted to serine, on the E-cadherin-luciferase reporter gene [38]. We confirmed that the wild type and the SUMO ligase mutant PIAS1 were expressed at equivalent levels (see Materials and Methods S1; Figures S2Ai and S2Aii). Importantly, we found that the SUMO ligase mutant PIAS1 failed to antagonize TGFβ-inhibition of the E-cadherin reporter gene (Figures 2Ai and 2Aii). We next carried out a dose-response study to further confirm the role and specificity of the SUMO ligase activity in the ability of PIAS1 to inhibit TGFβ repression of the E-cadherin promoter. These data showed that PIAS1 antagonized TGFβ-induced suppression of the E-cadherin reporter gene in a dose-dependent manner (Figure 2B). In contrast, the SUMO E3 ligase mutant PIAS1 (CS) did not block the ability of TGFβ to inhibit the E-cadherin reporter at any concentration (Figure 2B). Collectively, these data suggest that the SUMO ligase activity is critical for PIAS1 to block TGFβ-repression of E-cadherin transcription.

To characterize the role of endogenous PIAS1 on the ability of TGFβ to downregulate E-cadherin transcription, we employed an RNAi approach to induce knockdown of PIAS1 ([38] and Figures S2Bi and S2Bii). Expression of PIAS1 short hairpin RNAs enhanced the ability of TGFβ to suppress the E-cadherin promoter suggesting that endogenous PIAS1 counteracts TGFβ-suppression of E-cadherin reporter gene promoter activity (Figure 2C). Collectively, these data suggest that the SUMO E3 PIAS1 inhibits TGFβ-repression of E-cadherin expression.

**Figure 3:**
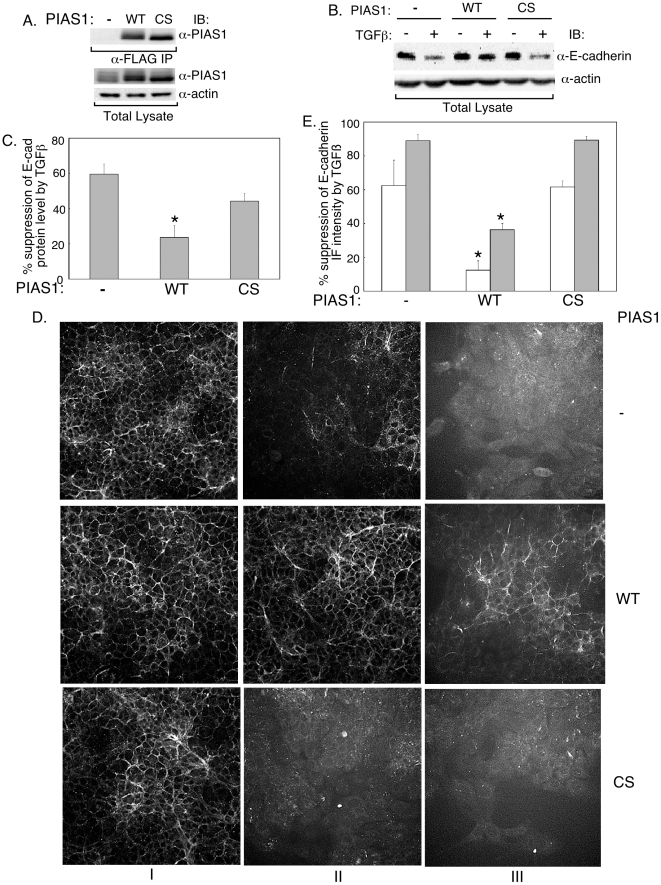
PIAS1 suppresses the loss of E-cadherin levels associated with TGFβ-induced EMT. A) Expression of wild type and the SUMO ligase mutant (CS) PIAS1 in stable NMuMG cells. Lysates of NMuMG cells stably transfected with the control vector expressing the resistance marker alone or together with wild type (WT) or SUMO E3 ligase mutant (CS) PIAS1 were subjected to anti-FLAG immunoprecipitation (α-FLAG IP) followed by anti-PIAS1 immunoblotting (upper panel), or were immunoblotted for PIAS1 and actin, the latter to serve as loading control (lower two panels). Wild type and SUMO ligase mutant PIAS1 (CS) are expressed at similar levels. B and C) PIAS1 acts in a SUMO E3 ligase dependent manner to suppress TGFβ-reduction in E-cadherin protein level. B) Lysates of NMuMG cells stably transfected with the control plasmid or a plasmid encoding WT or CS PIAS1 as in A and left untreated or incubated with TGFβ for 48 h were subjected to E-cadherin and actin immunoblotting. Scans are from a representative experiment that was repeated 4 times. C) Each column is the mean (± SEM, n = 4) of percent reduction by TGFβ of actin-normalized E-cadherin levels relative to the basal levels of the respective transfectant obtained from immunoblots including those shown in B (see MATERIALS and METHODS). D) Representative E-cadherin immunofluorescence images of vector control (−), wild type (WT) or SUMO E3 ligase mutant (CS) PIAS1 expressing NMuMG stable cells that were left untreated (I) or incubated with 20 pM (II) or 100 pM (III) TGFβ for 48 h. E) Quantification of E-cadherin immunofluorescence from images as shown in D. Intensity of E-cadherin immunofluorescence per cell was obtained and analyzed (see MATERIALS and METHODS). Each column is the mean (± SEM, n = 4) of percent reduction in E-cadherin levels by 20 pM (clear column) or 100 pM (grey column) TGFβ. * indicates statistical significant difference (p<0.05) as compared to the vector control.

We next used biochemical and cell-based approaches to test the possibility that PIAS1 negatively regulates TGFβ-induced EMT. We first generated NMuMG cells that stably expressed the wild type or SUMO ligase mutant PIAS1, or that were stably transfected with the control vector. Immunoblotting analyses confirmed that the wild type and SUMO E3 ligase mutant PIAS1 were expressed at equivalent level in these cells (Figure 3A). Comparison of the level of exogenous PIAS1 in these cells relative to control transfectants showed that expression of the wild type or SUMO E3 ligase mutant PIAS1 was only modestly increased relative to endogenous levels of PIAS1 (Figure 3A). Immunoblotting analyses using an antibody to E-cadherin revealed that expression of PIAS1 decreased the ability of TGFβ to suppress the levels of E-cadherin protein in NMuMG cells (Figure 3B). Quantitative analysis confirmed that the ability of PIAS1 to reverse TGFβ downregulation of E-cadherin levels was statistically significant (Figure 3C). In contrast, we found that mutation of the SUMO ligase activity significantly mitigated the ability of PIAS1 to inhibit TGFβ-repression of E-cadherin protein level (Figures 3B and 3C). Together, these data show that PIAS1 protects E-cadherin protein from TGFβ-dependent downregulation.

We also examined the effect of PIAS1 expression on different EMT parameters using cell-based assays. At the basal epithelial state, expression of PIAS1 did not appreciably affect the localization or expression of E-cadherin (Figure 3D). Similarly, we found that wild type or SUMO ligase mutant PIAS1 did not alter significantly the cortical actin organization or cuboidal cell shape in untreated cells (Figures S3A and S3B). In contrast, expression of wild type but not the SUMO ligase mutant PIAS1 drastically inhibited the ability of two different concentrations of TGFβ to induce a loss in E-cadherin levels (Figures 3D and 3E). Expression of PIAS1 suppressed the ability of TGFβ to induce the formation of actin stress fibers and fibroblastic cell phenotype (Figures S3A and S3B). In contrast, the SUMO ligase mutant PIAS1 did not reduce the ability of TGFβ to change actin reorganization and cell shape (Figures S3A and S3B). Collectively, these findings suggest that the SUMO E3 ligase activity of PIAS1 plays a critical role in suppressing the ability of TGFβ to induce EMT.

### The PIAS1 substrate SnoN contributes to PIAS1 inhibition of EMT

**Figure 4:**
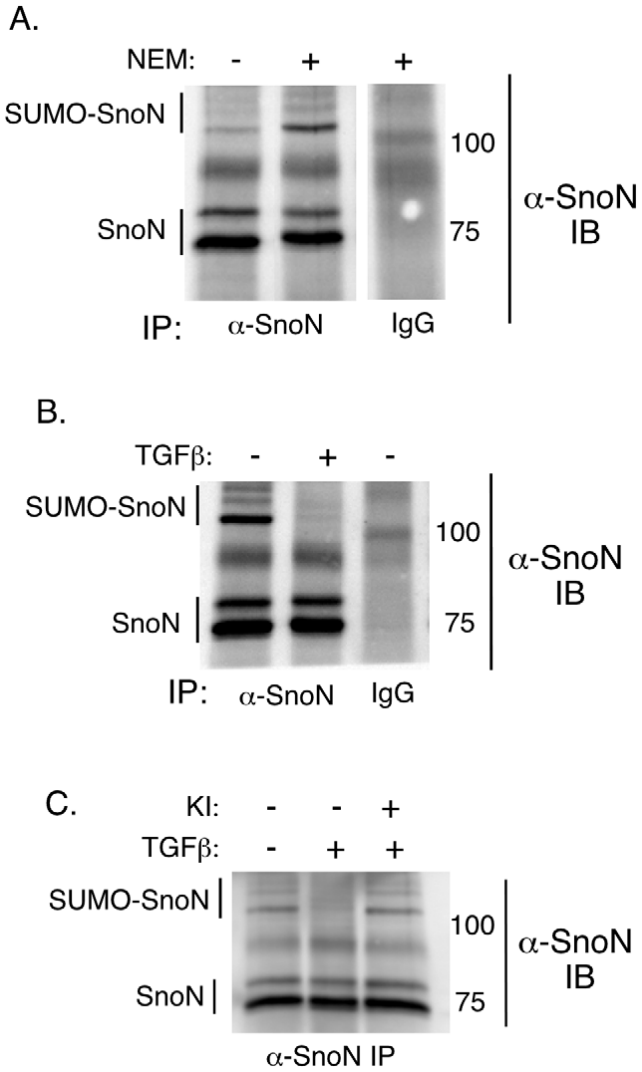
TGFβ inhibits SnoN sumoylation in cells undergoing EMT. A) Endogenous SnoN is sumoylated in NMuMG cells. NMuMG cell extracts prepared in the absence (−) or presence (+) of the isopeptidase inhibitor N-ethylmaleimide (NEM) were subjected to anti-SnoN or control IgG immunoprecipitation followed by SnoN immunoblotting. Several NEM-sensitive slow migrating SnoN-immunoreactive species as compared to unmodified SnoN were identified (SUMO-SnoN). B) TGFβ reduces SnoN sumoylation in cells undergoing EMT. Cells were left untreated or incubated with TGFβ for 48 h to induce EMT. NEM-containing lysates were analyzed as described in A. C) Inhibition of TβRI-dependent signaling blocks downregulation of SnoN sumoylation by TGFβ. NEM-treated lysates of NMuMG cells left untreated, or incubated with TGFβ alone or together with the TβRI inhibitor (KI) were subjected to SnoN immunoprecipitation followed by SnoN immunoblotting as described in A. Each of the scans in A, B and C is a representative blot from an experiment that was repeated two to three times.

The finding that the SUMO E3 ligase activity is required for the ability of PIAS1 to antagonize EMT suggested that specific SUMO substrates might mediate the effect of PIAS1 on this fundamental cellular process. The transcriptional regulator SnoN plays important roles in TGFβ signaling and responses, including antagonizing TGFβ-induced EMT in cancer cells [39], [49]. Recently, we and others showed that SnoN is sumoylated and that Lysines 50 and 383 on SnoN are major sites for SUMO conjugation [38], [50]. We also identified SnoN as a PIAS1 SUMO substrate [38], [50]. We therefore tested the hypothesis that sumoylation contributes to the ability of SnoN to inhibit TGFβ-induced EMT and that TGFβ triggers a reduction in the levels of PIAS1 and hence sumoylated SnoN to mediate EMT. SUMO-conjugated proteins can be difficult to detect due to their desumoylation by SUMO-proteases that might be activated during lysis [37], [51]. The general SUMO-protease inhibitor N-ethylmaleimide (NEM) is typically included in the lysis buffer to preserve the SUMO-conjugated species in the cellular extracts [52], [53]. Using SDS-PAGE analysis, the apparent molecular mass of these NEM-sensitive SUMO-conjugated species is 20 kDa or more as compared to the unmodified protein [37], [54]. We prepared NMuMG cellular extracts in the absence or presence of NEM and subjected these lysates to SnoN immunoprecipitation followed by SnoN immunoblotting. We first confirmed that endogenous SnoN was sumoylated in NMuMG cells as indicated by the appearance of several specific slow migrating NEM-sensitive SnoN immunoreactive species as compared to unmodified SnoN in SnoN immunoprecipitates (Figure 4A). Next, we examined the status of SnoN sumoylation in cells undergoing EMT. Interestingly, we found that TGFβ stimulation led to a reduction in the proportion of NEM-sensitive sumoylated-SnoN species in cells undergoing EMT (Figure 4B). In other experiments, inhibition of TβRI blocked the ability of TGFβ to reduce sumoylated-SnoN in these cells (Figure 4C). These data suggest that TGFβ signaling reduces the level of SnoN sumoylation in cells undergoing EMT.

**Figure 5:**
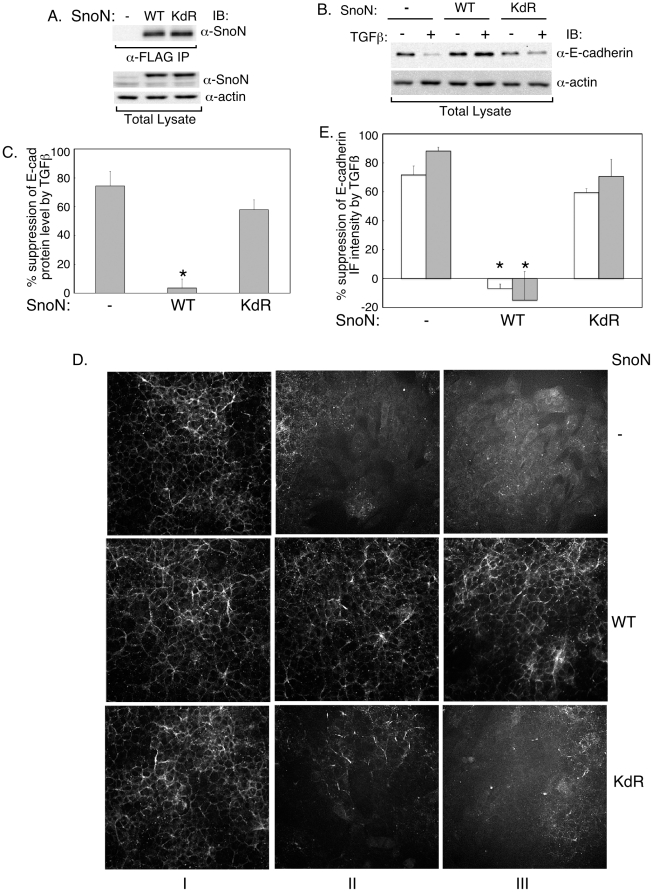
SnoN sumoylation contributes to the ability of SnoN to antagonize TGFβ-suppression of E-cadherin. A) Wild type SnoN and the SUMO loss of function SnoN (SnoN (KdR)) are expressed at equivalent levels in NMuMG stable cells. Lysates of NMuMG cells stably transfected with the control vector expressing the resistance marker alone or together with wild type SnoN (WT) or SUMO loss of function SnoN (KdR) were subjected to FLAG immunoprecipitation followed by SnoN immunoblotting (upper panel), or were immunoblotted for SnoN and actin, the latter serving as a loading control (lower two panels). B and C) Wild type but not the SUMO loss of function SnoN inhibits TGFβ-reduction in E-cadherin protein level in cells undergoing EMT. B) Representative scans of E-cadherin and actin immunoblots of lysates of control vector, wild type SnoN (WT) or SUMO loss of function SnoN (KdR) expressing cells that were left untreated or incubated with TGFβ. C) The densities of E-cadherin and actin immunoblots as shown in Figure 5B were analyzed (see MATERIALS and METHODS). Each column in the bar graph represents the mean (± SEM, n = 4) of percent suppression of E-cadherin protein level by TGFβ. D) Representative micrographs of E-cadherin-indirect immunofluorescence of vector control (−), wild type (WT) or SUMO loss of function (KdR) SnoN expressing NMuMG cells that were left untreated (I) or incubated with 20 pM (II) or 100 pM (III) TGFβ for 48 h. E) Intensity of E-cadherin immunofluorescence per cell was obtained and analyzed (see MATERIALS and METHODS). Each column in the bar graph represents the mean (± SEM, n = 3) of percent reduction in E-cadherin levels by 20 pM (clear column) or 100 pM (grey column) TGFβ. * indicates statistical significant difference (p<0.05) as compared to the vector control.

The finding that TGFβ decreases SnoN sumoylation suggested that this modification may contribute to SnoN's ability to inhibit EMT. To test this possibility, we compared the effect of stable expression of wild type SnoN or a SUMO loss of function SnoN mutant, in which Lysines 50 and 383 were converted to arginine residues (SnoN (KdR)), on EMT [38]. Wild type and mutant SnoN were expressed at equivalent levels (Figure 5A). Upon immunoblotting, we found that wild type SnoN inhibited the ability of TGFβ to suppress E-cadherin expression (Figures 5B and 5C). In contrast, expression of the SUMO loss of function SnoN mutant (KdR) had little or no effect on the ability of TGFβ to reduce E-cadherin expression (Figures 5B and 5C). Likewise, expression of wild type SnoN but not SnoN (KdR) antagonized TGFβ-inhibition of E-cadherin in the NMuMG cells as determined by immunocytochemistry (Figures 5D and 5E). These data suggest that sumoylation may be important for SnoN to inhibit TGFβ-induced EMT.

**Figure 6:**
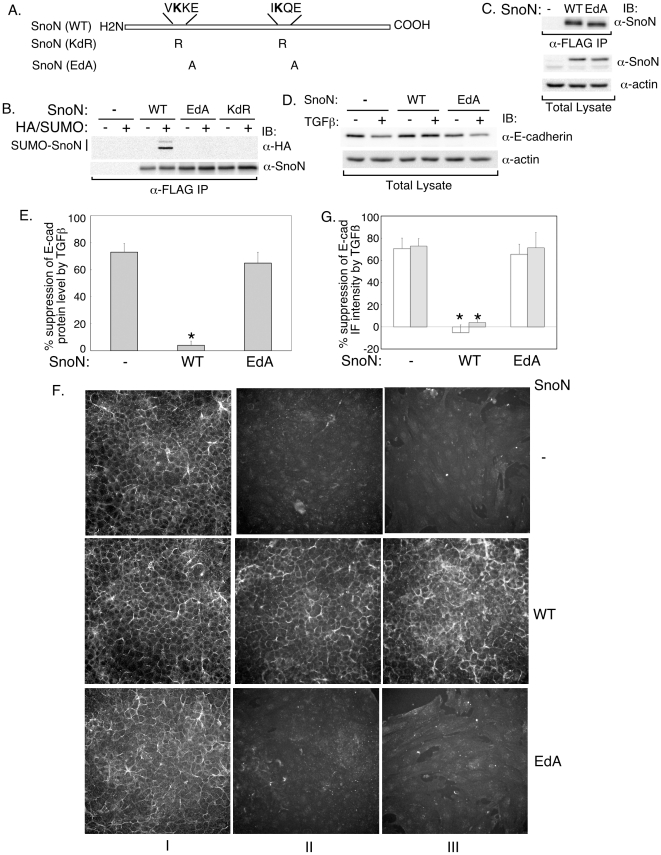
Sumoylation mediates the ability of SnoN to antagonize TGFβ-suppression of E-cadherin expression. A) A schematic showing the two SUMO consensus motifs in SnoN and the amino acid residues that are mutated in each of the SnoN (KdR) and SnoN (EdA). B) Glutamates 52 and 385 are critical for SnoN to be sumoylated on Lysines 50 and 383. NEM-treated lystaes of 293T cells expressing HA-tagged SUMO1, alone or together with SnoN (WT), SnoN (EdA) or SnoN (KdR) were subjected to FLAG immunoprecipitation followed by sequential HA (α-HA) and SnoN immunoblotting. Representative scans from an experiment that was done 4 times. C) Equivalent expression of SnoN (WT) and the SnoN (EdA) in NMuMG stable cells. Lysates of cells stably transfected with the control vector, SnoN (WT) or SUMO loss of function SnoN (EdA) were subjected to FLAG immunoprecipitation followed by SnoN immunoblotting, or were immunoblotted for SnoN and actin. D and E) SnoN (WT) but not SnoN (EdA) inhibits TGFβ-reduction in E-cadherin protein level in cells undergoing EMT. D) Scans of E-cadherin and actin immunoblots of lysates of control vector, SnoN (WT) or SnoN (EdA) expressing cells that were left untreated or incubated with TGFβ as in Figure 3B. E) Each column is the mean (± SEM, n = 5) of percent suppression of E-cadherin protein by TGFβ obtained by analysis of immunoblots including those shown in 6D (see MATERIALS and METHODS). F) Representative images of E-cadherin immunofluorescence of vector control (−), SnoN (WT) or SnoN (EdA) expressing NMuMG cells that were left untreated (I) or incubated with TGFβ (II and III) as in Figure 3D. G) Each column is the mean (± SEM, n = 5) of percent reduction in E-cadherin levels by 20 pM (clear column) or 100 pM (grey column) TGFβ from images as shown in 6F (see MATERIALS and METHODS). * indicates statistical significant difference (p<0.05) as compared to the vector control.

In addition to sumoylation, lysine residues can undergo other modifications including acetylation and methylation [37], [55]. To exclude the possibility that the lack of effect of the SnoN (KdR) on E-cadherin suppression by TGFβ is due to impairment of potential modifications other than sumoylation, we generated a distinct SUMO loss of function SnoN mutant (SnoN (EdA)) in which Glutamates 52 and 385 within SUMO consensus motifs (ψKXE) in SnoN were converted to alanine residues (Figure 6A). The acidic amino acid in position +3 of the SUMO consensus motif is critical for covalent linkage of the lysine residue with SUMO [37]. Accordingly, we found that replacement of Glutamates 52 and 383 with alanines abrogated the ability of SnoN to undergo sumoylation at Lysines 50 and 383 (Figure 6B).

We next compared the effect of stable expression of wild type and the SUMO loss of function SnoN (EdA) on EMT. Wild type SnoN and the SnoN (EdA) mutant were expressed at comparable levels in these cells (Figure 6C). Immunoblotting analyses showed that while wild type SnoN blocked the ability of TGFβ to reduce E-cadherin expression, the SnoN (EdA) mutant was ineffective in counteracting the TGFβ response (Figures 6D and 6E). Likewise, immunofluorescence studies showed that although wild type SnoN blocked downregulation of E-cadherin in NMuMG cells undergoing TGFβ-induced EMT, the SnoN (EdA) mutant failed to inhibit the TGFβ response (Figures 6F and 6G). Collectively, our findings suggest that sumoylation contributes significantly to the ability of SnoN to inhibit TGFβ-reduction of E-cadherin and hence induction of EMT. In turn, TGFβ decreases the level of sumoylated SnoN to facilitate EMT.

**Figure 7:**
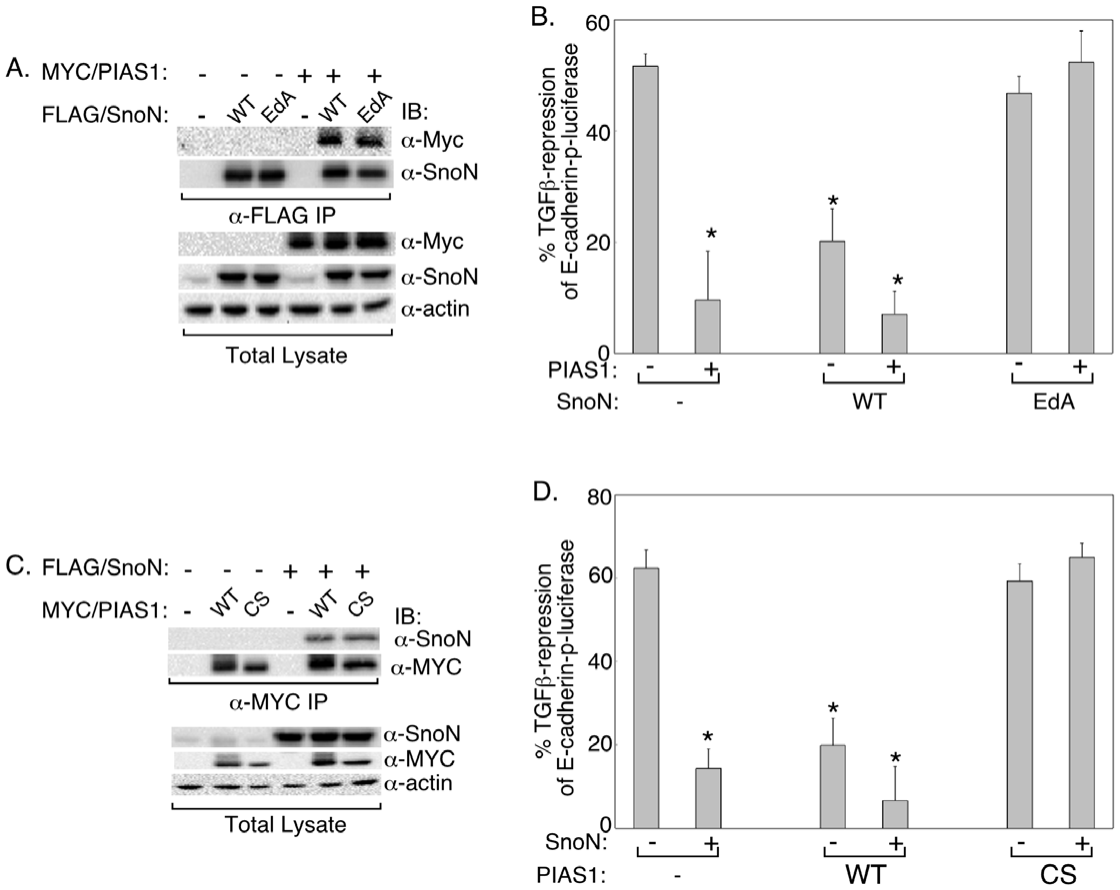
The PIAS1-SnoN sumoylation pathway antagonizes TGFβ-suppression of E-cadherin promoter activity. A) SUMO loss of function SnoN associates with PIAS1. Lysates of 293T cells expressing PIAS1, SnoN (WT) or SUMO loss of function SnoN (EdA) mutant, alone or together were subjected to SnoN immunoprecipitation (α-FLAG IP) followed by sequential PIAS1 (α-MYC) and SnoN (α-SnoN) immunoblotting. Expression of PIAS1, SnoN, and actin in lysates were confirmed by MYC, SnoN, and actin immunoblotting. B) The SUMO loss of function SnoN mutant inhibits PIAS1 to block TGFβ-reduction of E-cadherin transcription. NMuMG cells stably expressing SnoN (WT) or SnoN (EdA) or stably transfected with the vector control plasmid (−) were transiently transfected with the E-cadherin-p-luciferase reporter and the β-gal plasmid together with an empty expression vector (−) or one encoding the PIAS1 protein (+) were subjected to luciferase and β-galactosidase assays (see MATERIALS and METHODS). Each column is the mean (± SEM, n =  6) of percent reduction of E-cadherin-p-luciferase activity by TGFβ. C) The SUMO ligase mutant PIAS1 interacts with SnoN. Lysates of 293T cells expressing SnoN, PIAS1 (WT) or SUMO E3 ligase mutant PIAS1 (CS), alone or together were subjected to PIAS1 immunoprecipitation (α-MYC IP) followed by SnoN (α-SnoN) and PIAS1 (α-MYC) immunoblotting. Total lysates were immunoblotted as described in 7A. D) SUMO E3 ligase mutant PIAS1 reverses the effect of SnoN to inhibit TGFβ-repression of E-cadherin promoter activity. NMuMG cells stably expressing PIAS1 (WT) or PIAS1 (CS), or stably transfected with the vector control plasmid (−) were transiently transfected with the E-cadherin-p-luciferase reporter and the β-gal plasmid together with an empty expression vector (−) or one encoding the SnoN protein (+) were subjected to luciferase and β-galactosidase assays. Data from 6 independent experiments are presented as outlined in Figure 7B. * indicates statistical significant difference (P<0.05) as compared to vector control (column one of each B and D).

The finding that sumoylation contributes to SnoN's ability to inhibit TGFβ-induced EMT supports the conclusion that SnoN mediates the ability of the SnoN SUMO E3 ligase PIAS1 to suppress EMT. To further investigate the role of PIAS1-SnoN sumoylation pathway in the regulation of EMT, we examined the codependence of PIAS1 and SnoN in inhibiting TGFβ-induced downregulation of E-cadherin promoter. First, we asked whether expression of the SUMO loss of function SnoN (EdA) mutant might act in a dominantly interfering manner to block the function of the SUMO E3 ligase PIAS1 in NMuMG cells. Co-immunoprecipitation studies revealed that the SnoN (EdA) mutant was as effective as the wild type SnoN in associating with PIAS1 (Figure 7A). Importantly, in reporter analysis assays, we found that expression of the SnoN (EdA) mutant, but not the wild type SnoN, blocked the ability of PIAS1 to mitigate TGFβ repression of E-cadherin-promoter activity (Figure 7B). Quantitative immunofluorescence analysis showed that PIAS1 was expressed at similar levels in the control NMuMG cells as well as cells expressing wild type SnoN or the SnoN (EdA) mutant (Figures S4Ai and S4Aii). In other experiments, we examined the effect of expression of the SUMO E3 ligase inactive PIAS1 (CS) mutant on the ability of exogenous SnoN to block TGFβ-repression of E-cadherin promoter transcription. Co-immunoprecipitation experiments demonstrated that SnoN interacts with PIAS1 (CS) as effectively as with wild type PIAS1 (Figure 7C). In reporter assays, we found that expression of the PIAS1 (CS) mutant, but not the wild type PIAS1, reversed the ability of SnoN to counter TGFβ-repression of E-cadherin transcription (Figure 7D). Immunofluorescence analysis showed that SnoN was expressed at similar levels in control NMuMG cells as well as those expressing wild type PIAS1 or the PIAS1 (CS) mutant (Figures S4Bi and S4Bii). Together, these findings support the conclusion that SnoN plays a critical role in the ability of PIAS1 to block TGFβ-repression of E-cadherin promoter activity during EMT. Collectively, our findings point to an important functional link involving TGFβ, PIAS1 and SnoN in the regulation of EMT.

## Discussion

In this report, we identify a novel mechanism that regulates EMT. We have found that TGFβ reduces the levels of the SUMO E3 ligase PIAS1 in cells undergoing EMT. Expression of PIAS1 antagonizes the ability of TGFβ to induce EMT. Importantly, the SUMO E3 ligase activity of PIAS1 is critical for its suppression of EMT. TGFβ stimulation leads to the inhibition of sumoylation of the PIAS1 substrate SnoN. Loss of function mutations of SnoN sumoylation abrogated the ability of SnoN to inhibit TGFβ-induced EMT. Collectively, these data suggest that PIAS1-induced sumoylation of SnoN antagonizes EMT. Conversely, TGFβ leads to a reduction in the level of PIAS1 and sumoylated SnoN to facilitate the induction of EMT.

The identification of PIAS1 downregulation as a critical mechanism for TGFβ-induced EMT bears significant implications for our understanding of TGFβ responses. Since EMT comprises the loss of apical-basal polarity and cell-cell adhesion of epithelial cells, PIAS1 inhibition of EMT suggests that PIAS1 may play a critical role in maintaining cell polarity and cell-cell contact in epithelial tissues. In other words, the PIAS1 sumoylation pathway may operate as a checkpoint that must be overcome by TGFβ signaling in order for EMT to proceed. In future studies, it will be important to determine the role of PIAS1 activity in organizing the morphogenesis of epithelial tissues during development and in the control of metastasis of epithelial tumors. Because TGFβ regulates a diverse set of biological responses including cell proliferation, differentiation and apoptosis, our findings suggest that PIAS1 may also influence other TGFβ-regulated responses in addition to EMT.

PIAS1 interacts with a number of proteins including transcriptional regulators and signal transducers, many of which have been identified as PIAS1 SUMO substrates [46]. The finding that TGFβ triggers the downregulation of PIAS1 in cells undergoing EMT uncovers a novel mechanism that modulates PIAS1 actions. The increase in polyubiquitinated PIAS1 conjugates in cells undergoing EMT suggests that TGFβ signaling may lead to alteration in the expression or activity of components of the ubiquitin machinery that ultimately may regulate PIAS1 in these cells. It will be important in future studies to identify the ubiquitin ligases or deubiquitinases that may couple TGFβ signaling to the control of PIAS1 ubiquitination and consequent degradation.

An important question raised by our study is how PIAS1-induced sumoylation promotes the ability of SnoN to antagonize TGFβ-induced EMT. Sumoylation impacts the subcellular localization, stability, or transcriptional activity of protein substrates. However, sumoylation does not appear to affect the stability or subcellular localization of SnoN [38]. Therefore, it will be important in future studies to determine the molecular basis underlying the ability of sumoylation of SnoN to inhibit EMT.

In summary, our study defines an important and novel role for the SUMO E3 ligase PIAS1 as a suppressor of EMT. In particular, our data indicate that SnoN, a PIAS1 SUMO ligase substrate, might contribute to the ability of PIAS1 to inhibit EMT. On the other hand, TGFβ inhibits the PIAS1-SnoN sumoylation pathway in cells undergoing EMT. As EMT is critical for proper development and in the progression of cancer, it will be important in future studies to identify the role of PIAS1 and sumoylated SnoN in these biological processes.

## Materials and Methods

### Ethics Statement

N/A

### Plasmids

CMV-based plasmids to express FLAG-tagged wild type SnoN (WT), SUMO loss of function SnoN where Lysines 50 and 383 were converted to arginine residues (KdR), wild type PIAS1 (WT), and SUMO E3 ligase mutant PIAS1 where Cysteine 350 was converted to serine (CS) have been described previously [35], [38]. FLAG-tagged SUMO loss of function SnoN (EdA) in which Glutamates 52 and 385 were changed to alanines was generated by a PCR-based approach. The pCAGiP/FLAG expression vector containing cDNA encoding PIAS1 (WT or CS), or SnoN (WT, KdR, or EdA) protein was generated by subcloning the respective cDNA into the pCAGIP vector as described elsewhere [45], [56]. A pCAGiP vector enables the coexpression of the gene product of interest and the puromycin resistance marker from an internal ribosomal entry site (IRES) containing bicistronic transcript. The PIAS1 RNA interference vector encoding PIAS1 short hairpin RNAs and enhanced green fluorescent protein (GFP) under the control of the U6 and CMV promoters, respectively, was described previously [38], [57]. The E-cadherin-p-luciferase reporter, a generous gift from Dr. A. Cano, and the internal control β-galactosidase reporter constructs have been previously described [45], [47], [57]. The plasmids were confirmed by restriction digests and/or DNA sequence analyses (University of Calgary Core Sequencing Facility).

### Cell Cultures and Transfections

The NAMRU mouse mammary gland epithelial cells (NMuMG) were purchased from American Type Culture Collection (ATCC) and cultured in Dulbecco's modified Eagle's medium (Invitrogen) with high glucose and L-glutamine, supplemented with 10 mg/mL insulin and 10% fetal bovine serum (Invitrogen). The human kidney epithelial (293T) cells [58], [59] were maintained in Dulbecco's modified Eagle's medium (Invitrogen) with high glucose and L-glutamine, supplemented with 10% fetal bovine serum. NMuMG cells at approximately 70% confluency were incubated in regular media in the absence or presence of TGFβ and left at 37°C for 48 h to induce EMT. The TGFβ receptor type I kinase (TβRI) inhibitor, SB431542 (Sigma), was used at 10 μM, where indicated. 293T cells were transiently transfected using the calcium phosphate method. NMuMG cells were transiently transfected using FuGENE 6 (Roche Applied Science) or TransIT LT1 (Mirus) according to the manufacturer's instructions. Pools of NMuMG stables expressing a puromycin-resistant marker alone or together with PIAS1 or SnoN were obtained by transfecting cells with pCAGIP-based vectors containing specific cDNA using Lipofectin (Invitrogen) according to the manufacturer's instructions, followed by selection of resistant cells using 2 μg/ml puromycin (Invitrogen).

### Reporter Assays

NMuMG cells were seeded in 24-well plates at approximately 4.5×10^4^ cells/well one day prior to transfections. Cells were co-transfected with the E-cadherin-promoter-driven firefly luciferase reporter and the CMV-β-galactosidase internal control reporter constructs, together with a control expression vector or one encoding wild type PIAS1 (WT), SUMO ligase mutant PIAS1 (CS), or the PIAS1 RNAi plasmid (PIAS1i), as outlined in the figure legends. 18 h post transfection, cells were incubated in fresh media in the absence or presence of 100 pM TGFβ, and left for an additional 48 h, to allow for the induction of EMT where applicable. Lysates were prepared and analyzed for luciferase activity using a commercially available firefly luciferase assay kit [38], [45], [57]. Arbitrary luciferase activity (relative light units) values were normalized to β-galactosidase activity to account for variations in transfection efficiency. For each transfection, percent repression by TGFβ of E-cadherin promoter driven luciferase reporter gene expression was also determined and expressed relative to luciferase activity of the respective basal condition lysates (−TGFβ). Each experimental condition was carried out in triplicate, and the data presented represent the mean (± SEM) of five to six independent experiments.

### Reverse Transcription-PCR

NMuMG cells were lysed using TRIzol lysis reagent (Invitrogen), followed by total RNA extraction and concentration using chloroform and isopropanol/ethanol, respectively. The reverse transcriptase SuperScript II (Invitrogen) and oligo(dT)_12–18_ (Amersham Biosciences) as the primer were used to generate Poly(A)-cDNA as described before [38], [45], [57]. Mouse PIAS1 (1080 bp) and GAPDH (300 bp) cDNA fragments were PCR-amplified using the mouse poly(A)-cDNA as a template and gene specific PIAS1 (sense 5′-GGTATACGGGAAAAACCGG-3′; antisense 5′- TCAGAGGTTACGAGCAAAGG-3′), and GAPDH (sense 5′-CGGAGTCAACGGATTTGGTCGTAT-3′; antisense 5′-AGCCTTCTC CATGGTGGTGAAGAC-3′) oligonucleotides as primers. The PCR products were resolved and stained using 1.2% agarose gels and ethidium bromide, respectively. The amplified cDNA products were scanned and visualized using the VersaDoc 5000 Imager (Bio-Rad).

### Immunoprecipitation and Immunoblotting

NMuMG or 293T cell extracts prepared in TNTE (50 mM Tris-HCl, pH 7.4, 150 mM NaCl and 1 mM EDTA) buffer containing 0.5% Triton X-100 with protease and phosphatase inhibitors were centrifuged at 14,000 x *g* for 10 min at 4°C as described previously [38], [45], [57]. 20 mM N-ethylmaleimide (NEM; Sigma), an isopeptidase inhibitor, was included in the lysis buffer where indicated [38]. Supernatants' protein compositions were resolved by SDS-PAGE followed by western blotting using antibodies to proteins of interest. In experiments investigating PIAS1 ubiquitination or SnoN sumoylation, the majority of the supernatant was also subjected to immunoprecipitation using goat anti-PIAS1 (N-18; Santa Cruz), or rabbit anti-SnoN (H317; Santa Cruz) antibodies, respectively [38]. In studies examining the association of SnoN and PIAS1, a large fraction of the supernatant was subjected to immunoprecipitation using mouse anti-FLAG (M2; Sigma) or anti-MYC (9E10; Covance). Immunoprecipitations were performed on equivalent amounts of total protein in each sample. The protein contents of total cell lysate and immunoprecipitation samples were resolved by SDS-PAGE followed by immunoblotting using rabbit anti-SnoN, rabbit-anti ubiquitin (FL-76; Santa Cruz), mouse anti-E-cadherin (BD Transduction Laboratories), rabbit anti-PIAS1 (Epitomics), mouse anti-Smad2/3 (BD, Transduction Laboratories), rabbit anti-phospho Smad2 (Calbiochem), mouse anti-FLAG, mouse anti-MYC, and rabbit anti-actin (Sigma) as primary antibodies and horseradish peroxidase-conjugated anti-mouse or anti-rabbit antibody (Amersham Biosciences) as the secondary antibodies [57]. ECL (Amersham Biosciences)-generated signals were visualized and quantified using a VersaDoc 5000 Imager and Quantity One software (Bio-Rad), respectively.

In experiments investigating the effect of expression of wild type PIAS1, SUMO E3 ligase mutant PIAS1 (CS), wild type SnoN, or SUMO loss of function SnoN mutants on the ability of TGFβ to inhibit E-cadherin protein expression, the densities of E-cadherin and actin immunoblots in the absence and presence of TGFβ were quantified from appropriate scans including those shown in Figures 3B, 5B, and 6D, and E-cadherin levels were normalized to the corresponding actin levels. For each transfectant, reduction of actin-normalized E-cadherin protein levels by TGFβ was determined and expressed as a percent of actin-normalized E-cadherin levels in the respective basal (TGFβ-untreated) lysates. Each column in the bar graph shown in each of Figures 3C, 5C and 6E represents the mean (± SEM) of percent suppression of E-cadherin protein levels by TGFβ of a transfectant from several independent experiments as indicated by the n value in parenthesis in the respective figure legend.

### Immunocytochemistry and Fluorescence-Cell Based Analyses

1×10^4^ NMuMG cells were seeded per well in 96-well plates and incubated without or with TGFβ for 48 h to induce EMT. Cells were fixed with 4% formaldehyde, permeabilized with 0.2% Triton-X100, and blocked using 5% BSA and 5% calf serum in phosphate buffered saline (PBS) [45]. Subcellular localization and levels of E-cadherin were determined by incubating cells with a mouse anti-E-cadherin antibody followed by Cy3-conjugated anti-mouse IgG using a well established indirect immunofluorescence protocol [45]. For whole cell imaging, live cells were stained with 5 μM 5-chloromethylfluorescein diacetate (CMFDA) (Molecular Probes), according to manufactures instructions, followed by fixing as described above. For actin staining, fixed cells were incubated with TRITC-conjugated phalloidin (Sigma). All cells were incubated with the DNA fluorescent dye Hoechst 33342 (Invitrogen) to visualize their nuclei (data not shown). Images were acquired using the Cellomics Kinetic Scan Reader that is equipped with Carl Zeiss Axiom x microscope and a charge-coupled device (CCD) digital camera [45]. Representative E-cadherin immunofluorescence images are shown in Figures 1A, 3D, 5D, and 6F. Each micrograph corresponds to a 350 μm width. The SpotDetector bioapplication was used to quantify E-cadherin fluorescence intensity per cell, with cells identified by nuclear stain, from images including those shown in Figures 1A, 3D, 5D, and 6F. Each condition was carried out at least in triplicates per experiment, i.e. at least three wells of a 96-well plate, and the immunofluorescence data were averaged from a minimum of 2000 cells per well. For each condition, the reduction of E-cadherin immunofluorescence intensity by TGFβ was expressed as a percent of the E-cadherin immunofluorescence level in the respective basal (TGFβ-untreated) cells. Each column in each bar graph in Figures 1B, 3E, 5E, and 6G represents the mean (± SEM) of percent suppression of E-cadherin immunofluorescence intensity by TGFβ of a transfectant from several independent experiments as indicated by the n value in parenthesis in the respective figure legend.

### Statistical Analysis

Mean values of independent experiments repeated at least three times were subjected to student-t-test or analysis of variance (ANOVA) followed by post hoc tests to determine statistical significance (p<0.05).

## Supporting Information

Materials and Methods S1 (0.03 MB DOC)

Figure S1 Analysis of TGFβ-induced EMT and assessment of changes in levels of PIAS members by TGFβ. A and B) Analysis of TGFβ-induced EMT in NMuMG cells A) NMuMG cells left untreated, or incubated with TGFβ, the TβRI inhibitor SB431542 (KI), alone or together for 48 h were fixed and subjected to actin staining with TRITC-conjugated phalloidin. B) Cells treated as in A were incubated with the CMFDA whole cell fluorescent dye then fixed. Cells in A and B were also co-stained with the Hoechst DNA fluorescent dye (data not shown). Cells were scanned using the Cellomics KSR at X20 magnification (see MATERIALS and METHODS). Each micrographs shown in A and B represents 350 μm in width. C and D) PIAS 2, 3 and 4 may not be regulated during TGFβ-induced EMT. C) Transcript levels of PIAS family members in NMuMG cells may not be affected by TGFβ-induced EMT. RNA extracts from NMuMG cells that were left untreated or were incubated with TGFβ for 48 h, were subjected to reverse transcription followed by PCR amplification (RT-PCR) using specific primers for mouse PIAS1, PIAS2, PIAS3, PIAS4 and GAPDH, with the latter serving as internal control (see Material and Methods S1 for details). For each PIAS family member, 1 ng of an expression plasmid containing cDNA encoding the respective PIAS member was also subjected to PCR as a positive control (PCR). D) Determination of protein levels of PIAS2, PIAS3 and PIAS4 in NMuMG cells during EMT. Lysates of NMuMG cells left untreated or treated with TGFβ for 48 h, were immunoblotted for PIAS2, PIAS3 and PIAS4 and actin, the latter to serve as a loading control. Lysates of 293T cells transfected with control vector (−) or one expressing PIAS2, PIAS3 or PIAS4 (+) were subjected to the respective PIAS antibody immunoblotting as positive controls.
(5.05 MB TIF)

Figure S2 Quantitative analysis of PIAS1 levels in transiently transfected NMuMG cells. Ai) NMuMG cells transiently co-transfected with a GFP-expressing plasmid together with the control vector (−), or one encoding wild type (WT) or SUMO E3 ligase mutant (CS) PIAS1 cDNA were subjected to indirect immunofluorescence using an anti-PIAS1 antibody as primary antibody followed by incubations with Cy3-conjugated antibody, as the secondary antibody, and Hoechst 33342 nuclear stain. Cells were visualized using fluorescence microscopy for endogenous (−) or exogenous (WT or CS) PIAS1 (red), GFP (green) and nuclei (blue). Arrows indicate transfected cells as determined by GFP expression. Representative images of an experiment that was repeated five times show equivalent localization and expression of the wild type and SUMO E3 ligase mutant PIAS1 in transfected NMuMG cells. Aii) Transfected cells, as determined by GFP expression shown in Ai, were subjected to quantitative analysis of the intensity of the PIAS1 immunofluorescence signal (see Materials and Methods S1). Bi) Knockdown of endogenous PIAS1 in NMuMG cells by PIAS1 RNAi. NMuMG cells were transiently transfected with a control RNAi vector or one encoding a PIAS1 short hairpin RNA. Both RNAi vectors also co-expressed GFP. Cells were subjected to indirect anti-PIAS1 immunofluorescence and Hoechst 33342 nuclear staining, as in Ai, and were visualized using fluorescence microscopy for endogenous PIAS1 (red), GFP (green) and nuclei (blue). Scans are representative images from an experiment that was repeated four independent times. Arrows indicate transfected cells, as determined by GFP expression. Bii) Transfected cells, as determined by GFP expression shown in Bi, were subjected to quantitative analysis of the intensity of the PIAS1 immunofluorescence signal (see Materials and Methods S1 for details). Data show that PIAS1i is effective in reducing endogenous PIAS1 levels. Each column in graph shown in Aii and Bii represents the mean (± SEM) of the average PIAS1 intensity per cell from five and four independent experiments, respectively, expressed relative to the respective control. * indicates significant differences (P< 0.5) from the control.
(26.78 MB TIF)

Figure S3 PIAS1 suppresses the ability of TGFβ to induce actin reorganization and cell morphology change. A) Control vector (−), wild type PIAS1 (WT), or SUMO E3 ligase mutant PIAS1 (CS) expressing cells that were left untreated (I) or incubated with 20 pM (II) or 100 pM (III) TGFβ for 48 h were subjected to actin and nuclear fluorescent co-staining (see MATERIALS and METHODS). Wild type but not the SUMO E3 ligase mutant PIAS1 suppresses the ability of TGFβ to induce actin reorganization. B) Cells treated as in A, were costained with the whole cell fluorescent dye CMFDA and Hoechst 33342 nuclear stain (see MATERIALS and METHODS). Fluorescent images were captured as described in Figure S1. PIAS1 acts in a SUMO ligase dependent manner to reduce the ability of TGFβ to induce fibroblastic cell shape change associated with EMT.
(4.69 MB TIF)

Figure S4 Quantitative analysis of transiently expressed PIAS1 and SnoN levels in NMuMG cells. Ai) NMuMG cells stably expressing wild type SnoN (WT) or SUMO loss of function SnoN (EdA), or stably transfected with the vector control (−) were transiently transfected with a GFP-expressing plasmid together with an empty expression vector control (−), or one expressing the PIAS1 protein were subjected to indirect immunofluorescence using an anti-PIAS1 antibody as primary antibody followed by incubations with Cy3-conjugated antibody, as the secondary antibody, and Hoechst 33342 nuclear stain. Cells were visualized using fluorescence microscopy for endogenous (−) or exogenous PIAS1 (red), GFP (green) and nuclei (blue). Representative images of an experiment that was repeated four times show equivalent expression of exogenous PIAS1 in different stable NMuMG cells. Aii) Transfected cells, as determined by GFP expression shown in Ai, were subjected to quantitative analysis of the intensity of the PIAS1 immunofluorescence signal (see Materials and Methods S1 for details). Each column in the graph represents the mean (± SEM, n =  4 independent experiments) of the average exogenous PIAS1 intensity per cell of a stable transfectant expressed relative to its respective empty expression vector control. Bi) NMuMG cells stably expressing wild type PIAS1 (WT) or SUMO E3 ligase mutant PIAS1 (CS) or stably transfected with the vector control (−) were transiently transfected with a GFP-expressing plasmid together with an empty expression vector control (−), or one expressing SnoN protein were subjected to indirect immunofluorescence using an anti-SnoN antibody as primary antibody followed by incubations with Cy3-conjugated antibody, as the secondary antibody, and Hoechst 33342 nuclear stain. Cells were visualized using fluorescence microscopy for endogenous (−) or exogenous SnoN (red), GFP (green) and nuclei (blue). Representative images of an experiment that was repeated four times show equivalent expression of exogenous SnoN in different stable NMuMG cells. Aii) Transfected cells, as determined by GFP expression shown in Ai, were subjected to quantitative analysis of the intensity of the SnoN immunofluorescence signal (see Materials and Methods S1 for details). Each column in the graph represents the mean (± SEM, n =  4 independent experiments) of the average exogenous SnoN intensity per cell of a stable transfectant expressed relative to its respective empty expression vector control.
(14.20 MB TIF)
