# Supplementary material for: Suppression of TGFβ-Induced Epithelial-Mesenchymal Transition Like Phenotype by a PIAS1 Regulated Sumoylation Pathway in NMuMG Epithelial Cells
Source: PLoS One. 2010 Nov 12;5(11):e13971. doi: 10.1371/journal.pone.0013971 (PMC2980481; doi:10.1371/journal.pone.0013971)
Supplement: Materials and Methods S1 — (0.03 MB DOC) [file pone.0013971.s001.doc]

**Materials and Methods S1**

*Reverse Transcription (RT)-Polymerase Chain Reaction (PCR)*

RNA from NMuMG cells left untreated or incubated with TGFβ for 48 h was extracted and reverse transcribed using the oligonucleotide dT primer to produce polyA enriched complementary DNA (cDNA) (see MATERIALS and METHODS for details). cDNA fragments were PCR amplified using the mouse poly(A)-cDNA as a template and gene specific PIAS1 (see MATERIALS and METHODS for sequence of oligonucleotides), PIAS2 (sense 5’-CTATGAAAGTAACCAGCCAGC-3’; antisense 5’-CCCTTACAGAAGATGGCTGATAC-3’),PIAS3 (sense 5’-CATCAGAGGTTTGCCCCC-3’; antisense 5’-GTCAGGGCTCCTTTGCTTC-3’), and PIAS4 (sense 5’-CCAACATCGCTGTGAAGGTC-3’; antisense 5’-GGTTGGTGGCCGAGGACAG-3’) oligonucleotides as primers. 1 ng of expression plasmids containing cDNA encoding each of PIAS1, PIAS2, PIAS3 and PIAS4 were subjected to the corresponding PCR reaction as positive controls (Hsu et al 2006). GAPDH was amplified and used as an internal control (see MATERIALS and METHODS for details). Appropriate negative control PCR reactions were also performed. The PCR amplified products were resolved by ethidium-bromide-agarose gel electrophoresis and visualized by VersaDoc 5000 imager scanning.

*Immunoblotting*

Lysates of NMuMG cells left untreated or incubated with TGFβ for 48 h were subjected to SDS-PAGE and western blotting using goat-anti-PIAS2 (Santa Cruz), rabbit-anti-PIAS3 (Cell Signaling), goat-anti-PIAS4 (Santa Cruz), and rabbit anti-actin, the later used as a loading control. For each PIAS member analyzed, lysates of 293T cells transfected with the empty plasmid, or one encoding the respective PIAS family member were subjected to immunoblotting with the corresponding anti-PIAS antibody.

*Fluorescence microscopy imaging and quantification of immunofluorescence signal intensities*

NMuMG cells plated in 96-wells and transiently transfected with green fluorescent protein (GFP) expressing vector together with a control vector, SnoN expressing, PIAS1 expressing, or PIAS1 knockdown vector as described in the figure legends were fixed and subjected to indirect immunofluorescence (See MATERIALS and METHODS for details). Briefly, fixed cells were incubated with rabbit anti-SnoN or rabbit anti-PIAS1, followed by Cy3-fluorescently labelled anti-rabbit antibody, and Hoechst 33342 DNA fluorescent dye. The Cellomics Kinetic Scan Reader platform equipped with a Zeiss Axiome fluorescent microscope and a CDD camera was used to scan and generate images of fluorescently labelled cells at 20x magnification. We used the Target Activation BioApplication to determine the intensity of PIAS1-signal or SnoN-signal (red) in Hoechst stained NMuMG cells (blue) that were transfected, as identified by GFP expression (green). The Target Activation BioApplication, which accompanies the Cellomics KSR, is an automated image analysis algorithm that can quantify fluorescent intensities for multiple targets in individual cells. For each experiment, each transfection was carried out at least in duplicates, an average of more than 500 cells were scanned per well, intensity of the immunostained protein was normalized to number of cells in each replicate and averaged over replicates. Each experiment was repeated four to five independent times and ANOVA or Student t-test was used to determine statistical significance as compared to the control. The data in the bar graphs represent the mean (± SEM) of average SnoN or PIAS1 immunofluorescence intensity per cell expressed relative to that of the respective vector control transfection of four or five independent experiments (see Figure S2 and Figure S4 legends for specific details).
