# Supplementary figures and images for: Suppression of TGFβ-Induced Epithelial-Mesenchymal Transition Like Phenotype by a PIAS1 Regulated Sumoylation Pathway in NMuMG Epithelial Cells
Source: PLoS One. 2010 Nov 12;5(11):e13971. doi: 10.1371/journal.pone.0013971 (PMC2980481; doi:10.1371/journal.pone.0013971)

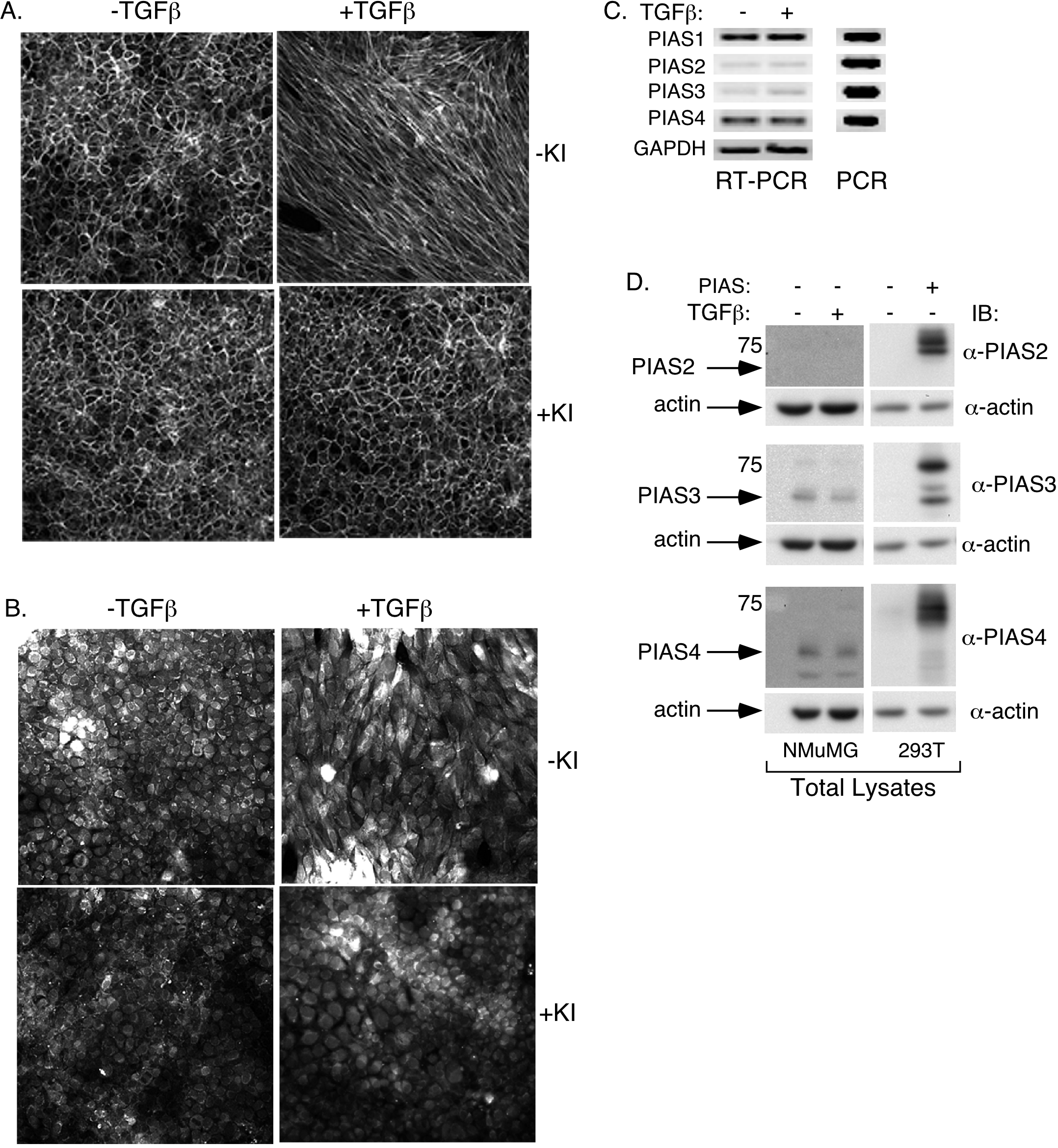

Supplement: Figure S1 — Analysis of TGFβ-induced EMT and assessment of changes in levels of PIAS members by TGFβ. A and B) Analysis of TGFβ-induced EMT in NMuMG cells A) NMuMG cells left untreated, or incubated with TGFβ, the TβRI inhibitor SB431542 (KI), alone or together for 48 h were fixed and subjected to actin staining with TRITC-conjugated phalloidin. B) Cells treated as in A were incubated with the CMFDA whole cell fluorescent dye then fixed. Cells in A and B were also co-stained with the Hoechst DNA fluorescent dye (data not shown). Cells were scanned using the Cellomics KSR at X20 magnification (see MATERIALS and METHODS). Each micrographs shown in A and B represents 350 μm in width. C and D) PIAS 2, 3 and 4 may not be regulated during TGFβ-induced EMT. C) Transcript levels of PIAS family members in NMuMG cells may not be affected by TGFβ-induced EMT. RNA extracts from NMuMG cells that were left untreated or were incubated with TGFβ for 48 h, were subjected to reverse transcription followed by PCR amplification (RT-PCR) using specific primers for mouse PIAS1, PIAS2, PIAS3, PIAS4 and GAPDH, with the latter serving as internal control (see Material and Methods S1 for details). For each PIAS family member, 1 ng of an expression plasmid containing cDNA encoding the respective PIAS member was also subjected to PCR as a positive control (PCR). D) Determination of protein levels of PIAS2, PIAS3 and PIAS4 in NMuMG cells during EMT. Lysates of NMuMG cells left untreated or treated with TGFβ for 48 h, were immunoblotted for PIAS2, PIAS3 and PIAS4 and actin, the latter to serve as a loading control. Lysates of 293T cells transfected with control vector (−) or one expressing PIAS2, PIAS3 or PIAS4 (+) were subjected to the respective PIAS antibody immunoblotting as positive controls. (5.05 MB TIF) [file pone.0013971.s002.tif]

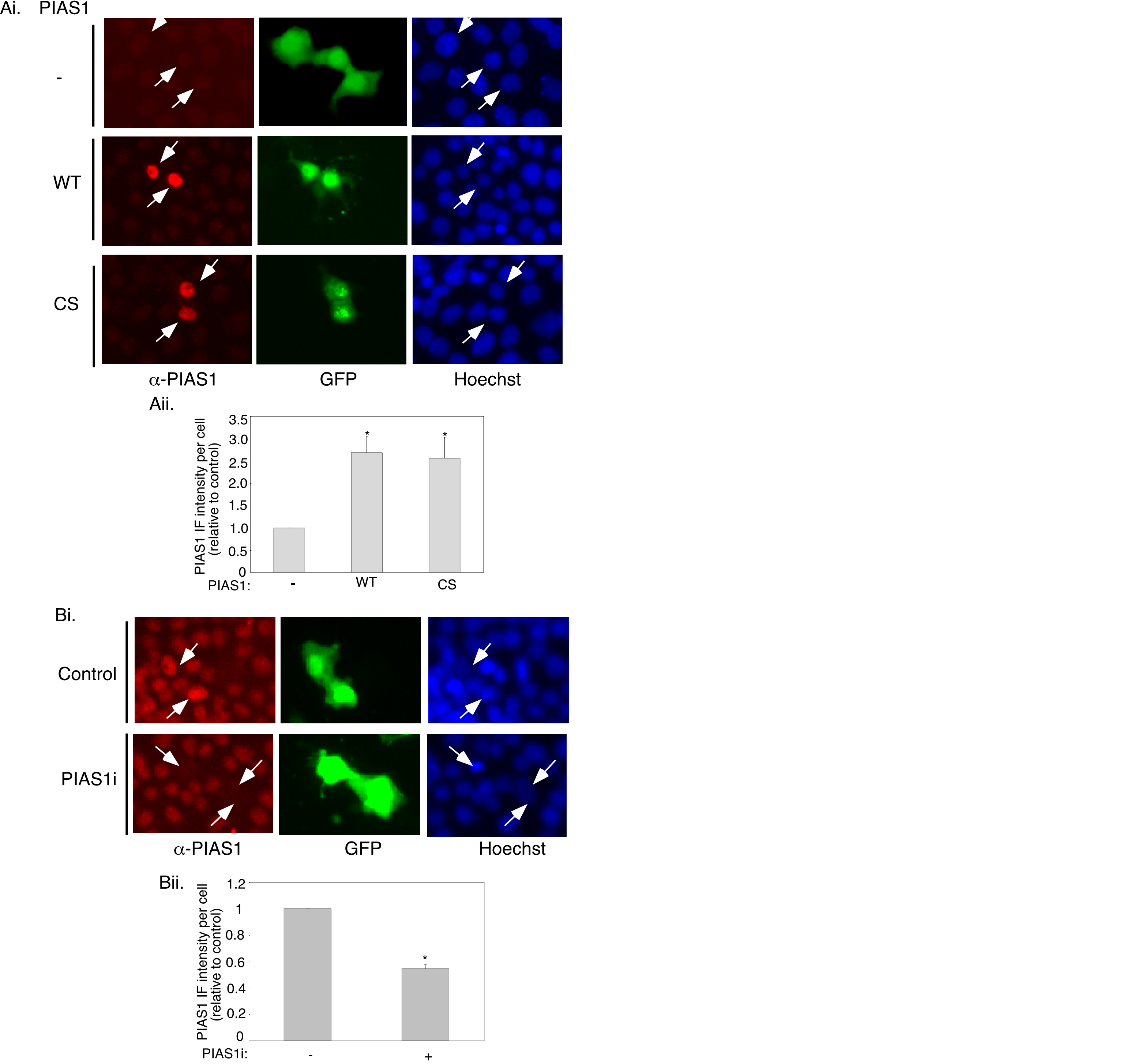

Supplement: Figure S2 — Quantitative analysis of PIAS1 levels in transiently transfected NMuMG cells. Ai) NMuMG cells transiently co-transfected with a GFP-expressing plasmid together with the control vector (−), or one encoding wild type (WT) or SUMO E3 ligase mutant (CS) PIAS1 cDNA were subjected to indirect immunofluorescence using an anti-PIAS1 antibody as primary antibody followed by incubations with Cy3-conjugated antibody, as the secondary antibody, and Hoechst 33342 nuclear stain. Cells were visualized using fluorescence microscopy for endogenous (−) or exogenous (WT or CS) PIAS1 (red), GFP (green) and nuclei (blue). Arrows indicate transfected cells as determined by GFP expression. Representative images of an experiment that was repeated five times show equivalent localization and expression of the wild type and SUMO E3 ligase mutant PIAS1 in transfected NMuMG cells. Aii) Transfected cells, as determined by GFP expression shown in Ai, were subjected to quantitative analysis of the intensity of the PIAS1 immunofluorescence signal (see Materials and Methods S1). Bi) Knockdown of endogenous PIAS1 in NMuMG cells by PIAS1 RNAi. NMuMG cells were transiently transfected with a control RNAi vector or one encoding a PIAS1 short hairpin RNA. Both RNAi vectors also co-expressed GFP. Cells were subjected to indirect anti-PIAS1 immunofluorescence and Hoechst 33342 nuclear staining, as in Ai, and were visualized using fluorescence microscopy for endogenous PIAS1 (red), GFP (green) and nuclei (blue). Scans are representative images from an experiment that was repeated four independent times. Arrows indicate transfected cells, as determined by GFP expression. Bii) Transfected cells, as determined by GFP expression shown in Bi, were subjected to quantitative analysis of the intensity of the PIAS1 immunofluorescence signal (see Materials and Methods S1 for details). Data show that PIAS1i is effective in reducing endogenous PIAS1 levels. Each column in graph shown in Aii and Bii represents the mean [file pone.0013971.s003.tif]

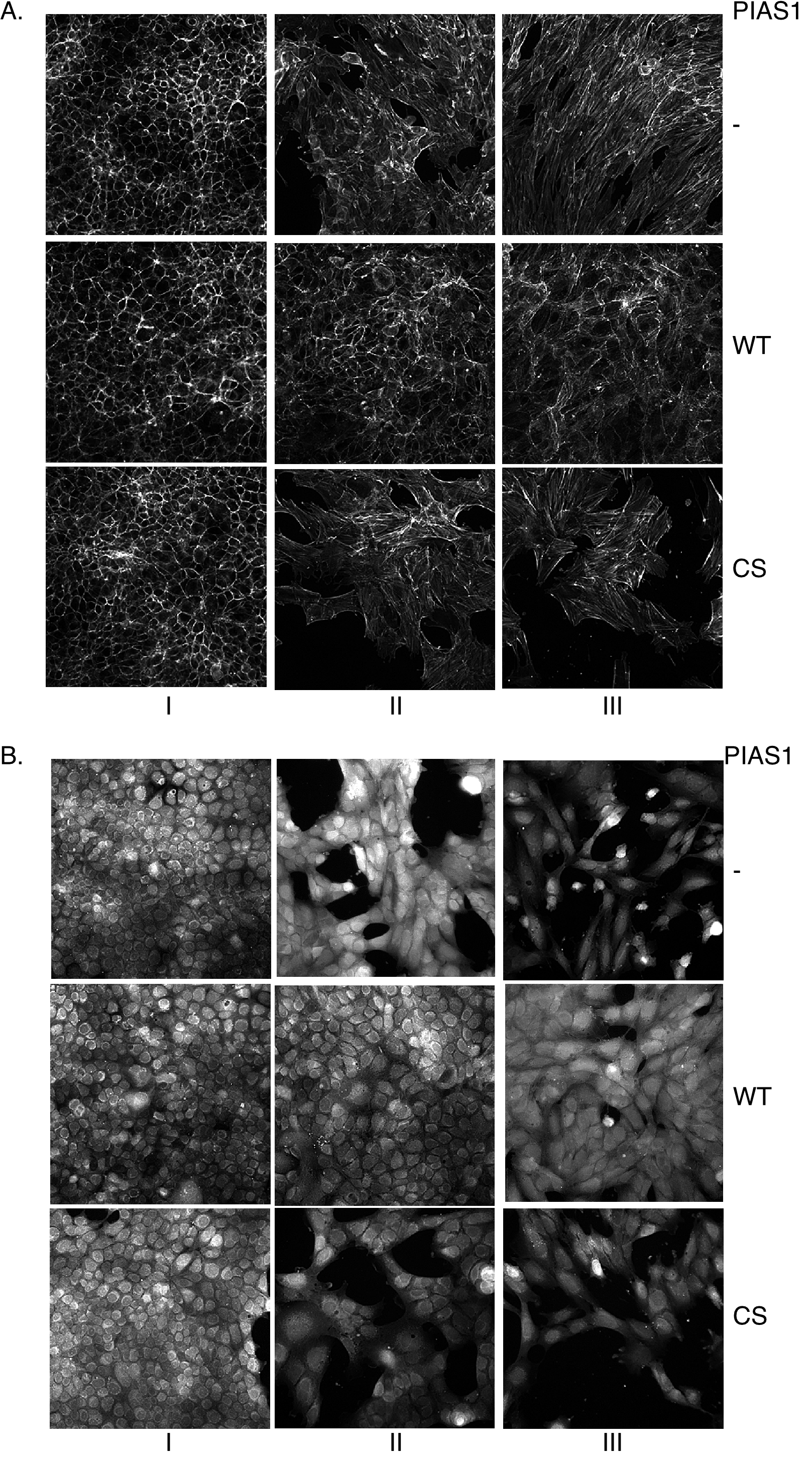

Supplement: Figure S3 — PIAS1 suppresses the ability of TGFβ to induce actin reorganization and cell morphology change. A) Control vector (−), wild type PIAS1 (WT), or SUMO E3 ligase mutant PIAS1 (CS) expressing cells that were left untreated (I) or incubated with 20 pM (II) or 100 pM (III) TGFβ for 48 h were subjected to actin and nuclear fluorescent co-staining (see MATERIALS and METHODS). Wild type but not the SUMO E3 ligase mutant PIAS1 suppresses the ability of TGFβ to induce actin reorganization. B) Cells treated as in A, were costained with the whole cell fluorescent dye CMFDA and Hoechst 33342 nuclear stain (see MATERIALS and METHODS). Fluorescent images were captured as described in Figure S1. PIAS1 acts in a SUMO ligase dependent manner to reduce the ability of TGFβ to induce fibroblastic cell shape change associated with EMT. (4.69 MB TIF) [file pone.0013971.s004.tif]

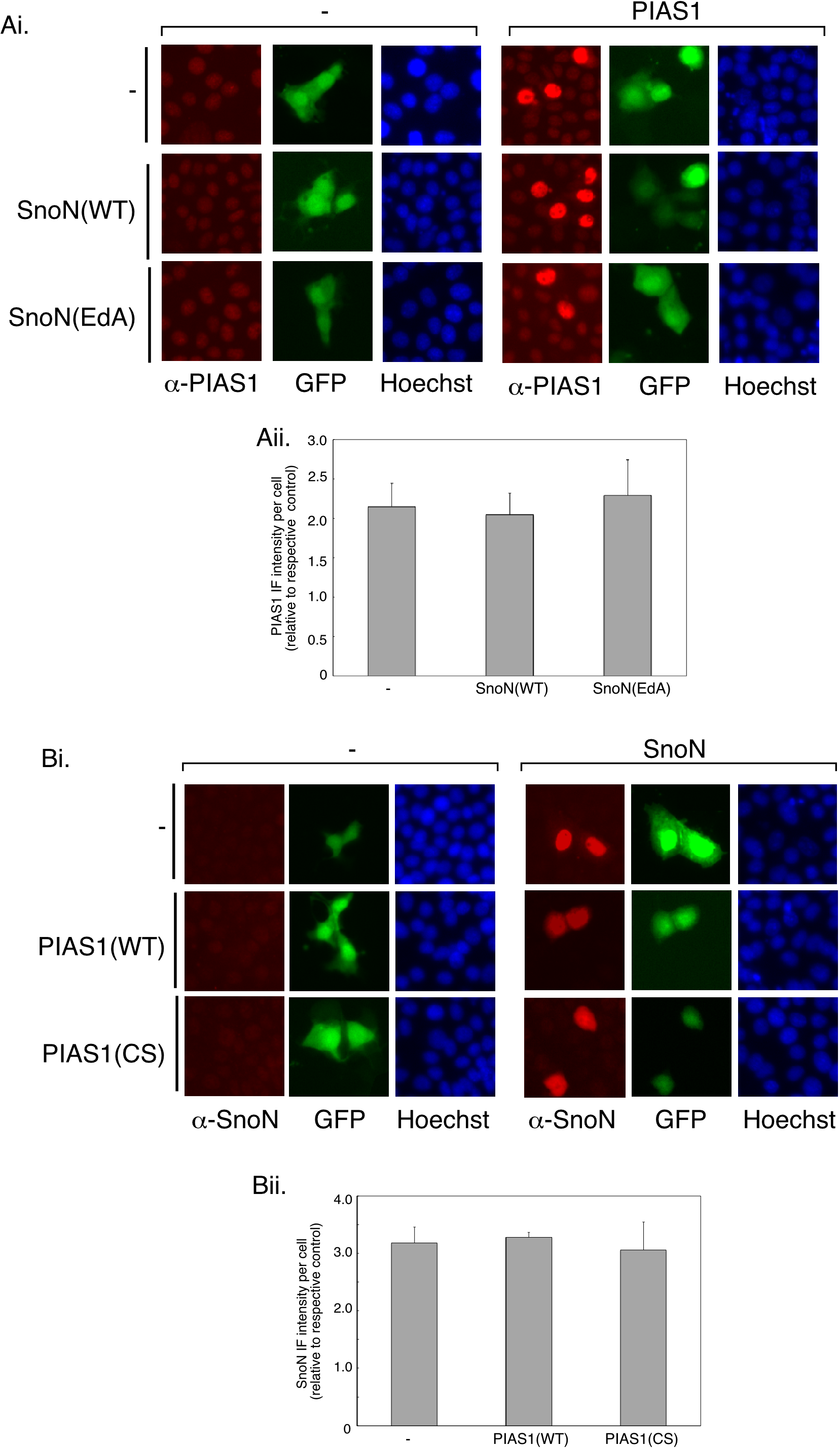

Supplement: Figure S4 — Quantitative analysis of transiently expressed PIAS1 and SnoN levels in NMuMG cells. Ai) NMuMG cells stably expressing wild type SnoN (WT) or SUMO loss of function SnoN (EdA), or stably transfected with the vector control (−) were transiently transfected with a GFP-expressing plasmid together with an empty expression vector control (−), or one expressing the PIAS1 protein were subjected to indirect immunofluorescence using an anti-PIAS1 antibody as primary antibody followed by incubations with Cy3-conjugated antibody, as the secondary antibody, and Hoechst 33342 nuclear stain. Cells were visualized using fluorescence microscopy for endogenous (−) or exogenous PIAS1 (red), GFP (green) and nuclei (blue). Representative images of an experiment that was repeated four times show equivalent expression of exogenous PIAS1 in different stable NMuMG cells. Aii) Transfected cells, as determined by GFP expression shown in Ai, were subjected to quantitative analysis of the intensity of the PIAS1 immunofluorescence signal (see Materials and Methods S1 for details). Each column in the graph represents the mean (± SEM, n = 4 independent experiments) of the average exogenous PIAS1 intensity per cell of a stable transfectant expressed relative to its respective empty expression vector control. Bi) NMuMG cells stably expressing wild type PIAS1 (WT) or SUMO E3 ligase mutant PIAS1 (CS) or stably transfected with the vector control (−) were transiently transfected with a GFP-expressing plasmid together with an empty expression vector control (−), or one expressing SnoN protein were subjected to indirect immunofluorescence using an anti-SnoN antibody as primary antibody followed by incubations with Cy3-conjugated antibody, as the secondary antibody, and Hoechst 33342 nuclear stain. Cells were visualized using fluorescence microscopy for endogenous (−) or exogenous SnoN (red), GFP (green) and nuclei (blue). Representative images of an experiment that was repeated four times show equivalent [file pone.0013971.s005.tif]
